# Childhood and adolescent obesity: Primary Health Care Physicians’ perspectives from Riyadh, Saudi Arabia

**DOI:** 10.12669/pjms.331.12118

**Published:** 2017

**Authors:** Faiza Nasser AlOtaibi, Majeedah AlOtaibi, Shiakhah AlAnazi, Hanan Al-Gethami, Deemah AlAteeq, Rowaydah Mishiddi, Amna Rehana Siddiqui

**Affiliations:** 1 Faiza Nasser AlOtaibi, Department of Family and Community Medicine, College of Medicine, King Saud University, Riyadh, Saudi Arabia; 2 Majeedah AlOtaibi, Department of Family and Community Medicine, College of Medicine, King Saud University, Riyadh, Saudi Arabia; 3 Shiakhah AlAnazi, Department of Family and Community Medicine, College of Medicine, King Saud University, Riyadh, Saudi Arabia; 4 Hanan Al-Gethami, Department of Family and Community Medicine, College of Medicine, King Saud University, Riyadh, Saudi Arabia; 5 Deemah AlAteeq, Department of Family and Community Medicine, College of Medicine, King Saud University, Riyadh, Saudi Arabia; 6 Rowaydah Mishiddi, Department of Family and Community Medicine, College of Medicine, King Saud University, Riyadh, Saudi Arabia; 7 Amna Rehana Siddiqui, Department of Community Health Sciences, Aga Khan University, Karachi, Pakistan

**Keywords:** Adolescent, Barriers, Childhood, Overweight, Obesity, Primary health care

## Abstract

**Background & Objectives:**

Primary health care (PHC) physicians are foremost to confront childhood and adolescent obesity. Our objective was to evaluate PHC Physicians perspectives for managing overweight/obesity in children and adolescents.

**Methods:**

PHC services from eight public hospitals in Riyadh participated. A self-administered tool maintaining anonymity evaluated facilitators and barriers for managing overweight/obese children and adolescent patients. Physicians who ‘always’ recommended weight management for an overweight / obese patient during past year, by involving patient, parents, and others were classified as having positive and appropriate practice.

**Results:**

Of the 58 respondents, 51.7% had appropriate practices. Lack of patient motivation (82.2%), and parental involvement (70.7%) were the major barriers. Physicians with appropriate practices differed in perspectives from those with less appropriate practices by attending continued education forums (p<0.026), referring patients to sub-specialty (p< 0.041), clinical knowledge (p<0.039), convinced on interventions (p<0.017), low concern for precipitating eating disorders (p<0.019), comfortable in examining obese patients (p<0.020), and considered patient’s readiness for weight change (p< 0.007).

**Conclusion:**

Efforts are needed to equip PHC physicians in managing overweight and obesity in Saudi children and adolescents.

## INTRODUCTION

Medical interventions combined with behavioral and environmental modifications address obesity and overweight status in children and adolescent.^1^ United States data (2012) report obesity prevalence of 18% in 6-11 and 21% in 12-19 years of age groups.^2^ Obesity prevalence is high across all age groups in Arab countries, attributed to urbanization, physical inactivity, prolonged sitting periods, fast food marketing, and other environmental factors.^3^,^4^ Increased body mass index (BMI) kg/m^2^ in children adversely affects cardiovascular risks^5^ and long-term consequences for impaired glucose tolerance, diminished quality of life and psychological problems.^6^,^7^

Obese children tend to become obese adults.^8^ Guidelines recommend Primary health care (PHC) physicians to identify obesity, medical risks, and unhealthy habits for eating and physical activity in children.^9^ Fundamental involvement of health professionals, parents, and children is further recommended.^9^-^11^ Lack of standardized training inhibits effective management of overweight and obesity.^11^ In Saudi Arabia prevalence of overweight, obesity, and severe obesity is 19.6%, 7.9%, and 1.5% in children, and 26.6%, 10.6%, and 2.4% in adolescent respectively.^12^

Gaps exist in PHC for training needs, inadequate practices, negative feelings, lack of resources, self-perceived treatment efficacy, and disconnect between recommended guidelines and practices.^13^-^21^ Such gaps in high risk settings like Saudi Arabia can undermine obesity control. We evaluated PHC physicians’ perspectives in managing childhood and adolescent obesity in Riyadh, Saudi Arabia.

## METHODS

PHC services of eight large hospitals working under health department of Riyadh, participated to provide PHC physicians’ perspectives on management of childhood/adolescent obesity. In March 2011, informed consent forms, self-administered questionnaires (12 per hospital) and a drop box were handed over to head of PHC clinics, and completed forms were collected after one week. Study tool was constructed using reference questions^13^-^19^ and was pretested. Study tool was coded for participants’ anonymity, inquired demographic, professional, and lifestyle factors. Perspectives on, initiating treatment, involving patients & parents, assessment methods, referred sub-specialties, and barriers at PHC level on a five-point scale using “always, mostly, sometimes, rarely, & never”. Two questions created ‘Appropriate practice’ variable; one “During past year how often you made recommendations for weight control when overweight/obese children/adolescents were identified”; those responding ‘always’ were coded as ‘one’; and those who responded ‘most of the time, sometimes, or rarely’ were coded as ‘two’. Second question, “Whom do you engage with routinely when you treat overweight/obese children and adolescent?”, those responding for all ‘patient, parents, and others” were coded ‘one’, and rest were coded as ‘two’. PHC Physicians’ with positive/appropriate practices were compared with those with less appropriate practices.

Sparse data in extremes of five-point scale ensued combining “always and most of the time” and “rarely and never” categories. Chi square was used as test of significance for categorical variables and a p value of <0.05 was considered significant.

## RESULTS

Overall 63.4% (58/92) physicians responded, 60.3% were males, and 52% worked for 10-40 hours per week. Fifty-three percent had < 10 years, 35.9% 10-20 years, and 12.1% had > 20 years of PHC experience. Mean BMI of physicians was 27.35 (SD 3.8) kg/m^2^, 65% were overweight, 42% exercised for > 3 days/week, and 41.3% consumed low-fat diet. More than 80% of physicians reported that patients rarely follow interventions. PHC Physicians’ demonstrated varied initiatives, assessment methods, information sources, and referral specialty (Table-I). Lack of, patient motivation, parental involvement and of support services were major barriers (Table-II). Physicians’ with appropriate practices (51.7%), differed by those with less appropriate practices on gender, attending workshops/seminars/CME, sub-specialty referral, considering patients’ readiness, examining obese patients comfortably; did not perceive lack of clinician knowledge, precipitation of eating disorders, and futility of interventions as barriers (p<0.05) (Table-III).

**Table-I:** PHC Physicians’ perspectives for managing overweight and obesity status.

How often do you initiate treatment in the following groups?	N	Always+Mostly % (n)	Some times %(n)	Rarely Never %(n)
A-Overweight children with no obesity-associated medical conditions.	56	33.9 (19)	48.2(27)	17.8(10)
B-Overweight adolescents with no obesity-associated conditions.	57	38.6(22)	47.4(27)	14.0(8)
C-Overweight children who do not want to control their weight.	57	43.9(25)	42.1(24)	14.0(8)
D-Overweight adolescents who do not want to control their weight.	57	43.9 (25)	43.9(25)	12.3(7)
How often recommended weight control/past year?	58	65.5 (38)	29.3(17)	5.1(3)
***Which of the following assessment methods were used?***				
A-Clinical impression.	54	74.1(40)	13.0(7)	13.0(7)
B-Weight for age percentile.	57	50.9(29)	45.6(26)	3.6 (2)
C-Weight for height percentile.	55	58.2(32)	34.5(19)	7.2(4)
D-Body mass indices	57	67.1(40)	5.3(3)	24.6(14)
E-Skin fold thickness percentile.	55	10.9(6)	23.6(13)	65.5(36)
F-Waist to hip ratio percentile or waist ratio percentile.	57	22.8(13)	35.1(20)	42.1(24)
***What information sources you use for assessing overweight and obesity?***				
Medical school/residency training	57	64.9(37)	22.8(13)	12.3(7)
Professional/journal articles	54	42.6(23)	31.5(17)	25.9(14)
Workshops/seminars/programs/CME programs	54	72.2(39)	18.5(10)	9.3(5)
Textbooks	56	76.7(43)	14.3(8)	8.9(5)
Experience	55	67.3(37)	29.1(16)	3.6(2)
Mass Media	53	33.9(18)	34(18)	32(17)
Computer programs/websites	55	52.7(29)	34.5(19)	12.7(7)
Pharmaceutical companies	52	15.4(8)	32.7(17)	51.9(27)
***Use of referral programs and specialists for overweight and obese patients***				
Behavior modification/behavior therapist	56	39.3(22)	32.1(18)	28.5(16)
Family therapy	57	39.6(22)	33.3(19)	28.0(16)
Group therapy	56	26.8(15)	32.1(18)	41.0(23)
Dietitian/Nutritionist	57	80.7(46)	12.3(7)	7.0(4)
Exercise specialist	55	43.6(24)	27.3(15)	29.0(16)
Child/Adolescent weight loss program	56	47.9(22)	27.6(16)	31.1(18)
Pediatric obesity specialist or program	57	52.6(30)	17.5(10)	29.8(17)
Pediatric subspecialist (endocrinology, pulmonology, orthopedic)	57	38.6(22)	40.4(23)	21.1(12)
Commercial adult weight loss program	52	15.4(8)	38.5(20)	46.1(24)
Self-help programs	57	35.1(20)	35.1(20)	29.8(17)
Camps for overweight children/adolescents	55	12.8(7)	14.5(8)	70.9(39)

**Table-II:** Barriers in managing childhood/adolescent obesity at PHC level.

How often is each of the following an important barrier?	N	Always+ Mostly % (n)	Some Times % (n)	Rarely+ Never % (n)
a-Lack of patient motivation and non-compliance	57	82.2(47)	15.8(9)	1.8 (1)
b-Lack of parent involvement in treatment	58	70.7(41)	27.6(16)	1.7 (1)
c-Lack of clinician time	56	41.1(23)	46.4(26)	12.5 (7)
d-Lack of reimbursement (No pay for the preventive services)	54	26.0(14)	40.7(22)	33.3(18)
e-Lack of clinician knowledge about treatment	57	12.3(7)	56.1(32)	31.6(18)
f-Lack of individual treatment skills	56	21.5(12)	64.3(36)	14.3 (8)
g-Lack of support services (e.g. nutrition, counseling)	56	51.8(29)	42.9(24)	5.3 (3)
h-Futility (feeling that interventions are ineffective)	55	20.0(11)	49.1(27)	30.9(17)
i-Concern about precipitating eating disorders	56	19.6(18)	25.0(20)	55.3(19)
j-I feel uncomfortable when examining an obese patient	56	19.6(11)	25.0(14)	55.3(31)

**Table-III:** PHC Physicians’ characteristics and practices for managing childhood obesity.

Characteristics	Positive Practices	Less positive Practices	p value
PHC Physician sex	N=30	N=27	
Males	46.7%	77.8%	0.028
Females	53.3%	22.2%	
Attended workshops/seminars/CME programs	N=27	N=26	
Always and Most of the time	85.2%	57.7%	0.026
Sometimes + Rarely + Never	14.8%	42.3%	
Referral to pediatric obesity specialist or program	N=29	N=27	
Always and often	65.5%	37.0%	0.041
Sometimes	6.9 %	39.6%	
Rarely and never	27.6%	33.3%	
Assess readiness to make changes to manage weight:	N=30	N=27	
Always and Most of the time	90.0%	59.3%	0.007
Sometimes	10.0%	40.7%	
Lack of clinician time	N=28	N=27	
Always and most of the time	57.1%	25.9%	0.055
Sometimes	35.7%	55.6%	
Rarely and never	7.1%	18.5%	
Lack of clinician knowledge about treatment	N=29	N=27	
Always and most of the time	6.9%	18.5%	0.039
Sometimes	48.3%	66.7%	
Rarely and never	44.8%	14.8%	
Futility (interventions are ineffective)	N=28	N=26	
Always and most of the time	17.9%	23.1%	0.017
Sometimes	35.7%	65.4%	
Rarely and never	46.4%	11.5%	
Concern about precipitating eating disorders	N=29	N=27	
Always and most of the time	44.8%	14.8%	0.019
Sometimes	20.7%	51.9%	
Rarely and never	34.5%	33.3%	
I feel uncomfortable when examining an obese patient	N=29	N=26	
Always and most of the time	20.7%	19.2%	0.020
Sometimes	10.3%	42.3%	
Rarely and never	69.0%	38.5%	

## DISCUSSION

More than half of PHC physicians displayed positive practices for managing childhood obesity through focusing on self-development to overcome reported barriers. This study demonstrates major roles of barriers as, lack of clinician’s knowledge and skills, poor patient motivation and limited parental involvement. A recent survey of 707 Saudi physicians identified training needs to manage obesity, especially in counseling for nutrition and physical activity.^22^ Likewise, a USA based study emphasized multidisciplinary weight management programs, where PHC Physicians mainly focused on, family, diet, physical activity, and behavior therapy.^23^ Expanding role of PHC in obesity management recommends to advance patient motivation, behavior and life style as standard practices.^24^ Patient readiness, parental involvement, and physicians’ knowledge are crucial for managing child/adolescent obesity.^11^,^15^,^21^ Patients’ and physicians’ views are reported to differ on weight management, providers perceived hopelessness on patient’s ability to lose weight, where-as patients’ were not convinced of role of medical care.^15^ In-depth interviewing of parents who either completed or withdrew from weight management program for their children showed the importance of ‘preparing the clinic’ and support services for tailored advice.^11^ Increased knowledge of family physicians increased discussion for weight loss, and recommendations to other services.^21^ Our findings are consistent with studies reporting lack of patients’ motivation, parental involvement and support services.^13^ Other known important barriers were related to time constraints, poor administrative support, and lack of sub-specialty groups for referral services.^13^-^14^,^20^,^25^ Our study also identified such factors and health services need multifaceted interventions to tackle childhood/adolescent overweight and obesity.^14^

Barriers described in our study have also been reported from Saudi Arabia^20^, Bahrain^14^, Israel^19^, France^16^, USA^13^,^21^, Canada^25^, and Australia.^18^ A nationally representative survey from USA identified self-reported low levels of counseling related skills for weight management by pediatric nurse practitioners, and pediatricians.^13^ Lack of both discipline and motivation in patients were considered negative attitudes from family physicians that could hinder the successful obesity management.^21^

Analogous to our finding, that patients rarely follow interventions, poor administrative support, lack of sub-specialty groups for referral services^13^-^14^,^20^ could translate in decline of obesity management.^14^ Our study showed more females than men having positive practices, though role of chance could not be excluded, but few studies report female physicians significantly referring children.^17^,^23^ A low response survey for assessment of practices and characteristics of pediatricians, dieticians, and nurse practitioners on management of overweight and obesity in children and adolescent showed a high response rate from female participants.^17^ Another study that examined family physicians and pediatricians for the factors influencing referral of obese adolescents to support services informed that female physicians significantly referred more patients than male physicians.^23^ A nationally representative study form Canada identified need for effective assessment tools, practice guidelines, treatment resources, enhancement of medical curriculum, and health system level changes to address childhood overweight and obesity management.^25^

Our study strengths included multicenter participation, representing various catchment areas, use of referenced questions,^13^-^19^ and respondent anonymity, reducing potential biases. The low response rate (60.4%) limits generalizability, however, several studies on similar theme reported response rates of less than 53%.^17^,^21^ Regarding our study if only a committed group responded, then non-responders possessed less appropriate practices, if those with appropriate practices did not participate, nonetheless, we would endorse our findings. Our results are consistent for specific training needs; information sources, patients’ readiness, and involvement of stakeholders. Longitudinal studies need to be designed on heterogenous assessment of obesity to evaluate weight changes accounting for pubertal and age related changes for evidence based interventions.

## CONCLUSION

Efforts are needed to equip PHC physicians for upgrading their knowledge, beliefs, and skills for addressing complex and multidimensional areas of childhood and adolescent overweight and obesity. PHC services in Saudi Arabia can play a crucial role in cultivating patient’s motivation and engaging parents effectively to identify, manage, and prevent childhood and adolescent overweight and obesity.
